# Relative devaluation in AI transitions: an organizational behavior perspective on career sustainability beyond well-being

**DOI:** 10.3389/fpsyg.2026.1822711

**Published:** 2026-06-23

**Authors:** Wei Zheng, Mohd Mohid Rahmat, Noor Hasni Juhdi, Mohd Fahmi Ghazali, Meng Na

**Affiliations:** 1 School of Auditing, Nanjing Audit University Jinshen College, Nanjing, China; 2 Faculty of Economics & Management, Universiti Kebangsaan Malaysia, UKM, Bangi, Selangor, Malaysia; 3 Graduate School of Business, Universiti Kebangsaan Malaysia, UKM, Bangi, Selangor, Malaysia

**Keywords:** AI governance, algorithmic management, algorithmic relative devaluation, career reconfiguration, career sustainability, metric governance

## Abstract

AI-enabled and digitally benchmarked evaluation systems are increasingly used in professional assessment, yet their implications for perceived professional value remain insufficiently understood. Drawing on Chinese higher education as a highly metricized institutional context, this study examines algorithmic relative devaluation (ARD), defined as professionals’ perception that their contribution is comparatively downgraded when algorithmic or AI-mediated standards become institutionally privileged. Using a three-wave time-lagged survey of faculty members (*N* = 448) and PLS-SEM analysis, the findings indicate that algorithmic visibility and benchmark intensity are positively associated with ARD, whereas algorithmic explainability is negatively associated with ARD. ARD is, in turn, associated with both adaptive and defensive career reconfiguration and with lower perceived career sustainability. Adaptive career reconfiguration is positively associated with perceived career sustainability, whereas defensive career reconfiguration is negatively associated with perceived career sustainability. The findings further suggest that responsible AI governance plays an ambivalent role: it weakens the devaluing association of algorithmic visibility but strengthens the devaluing association of benchmark intensity, while not significantly moderating the explainability–ARD relationship. Rather than demonstrating causal restructuring of professional hierarchies, the study offers evidence that perceived AI-enabled evaluation features are linked to comparative worth perceptions and divergent career-related responses in a metric-intensive academic setting. The findings contribute to research on algorithmic management and sustainable careers by positioning ARD as a context-specific mechanism through which AI-mediated evaluation may shape perceived professional value beyond job insecurity and well-being concerns.

## 1 Introduction

Professional evaluation in algorithmically intensive workplaces is no longer anchored exclusively in peer judgment or managerial discretion. Across sectors, AI-enabled and digitally benchmarked systems now generate performance indicators—citation dashboards, predictive scoring tools, algorithmic productivity monitors, and grant-ranking platforms—that are increasingly consequential for promotion, resource allocation, and professional recognition (Bond et al., 2024; McKinsey, 2024; Stanford Institute HCAI, 2024). Recent global assessments report widespread adoption of AI applications across analytics, monitoring, and decision-support functions. As these systems become institutionally embedded, they may function as computational reference standards through which professional competence is assessed and compared. This raises a question that existing scholarship has not fully addressed: when algorithmically privileged indicators become a dominant basis for institutional evaluation, how do professionals perceive the relative standing of their broader contribution?

Research on AI and work has documented heterogeneous labor-market and organizational effects. Labor analyses identify productivity gains and wage premiums in some high-skill roles alongside displacement and polarization risks in routine-intensive tasks (Chen et al., 2025; Njekwa Ryberg, 2026). Organizational studies link AI-enabled monitoring and algorithmic management to technostress, emotional exhaustion, job insecurity, and burnout, particularly under continuous digital oversight (Hamouche et al., 2023; Kumari and Tiwari, 2026; Xiong et al., 2025). In healthcare, large-scale validation studies show AI diagnostic models in radiology and oncology matching or exceeding specialist-level accuracy in specific tasks, further reinforcing algorithmic benchmarks as reference points for expertise (Bian et al., 2025; Huang et al., 2025). These contributions establish that AI evaluation systems can generate material threat, surveillance pressure, and psychological strain. However, they primarily frame consequences in terms of displacement, control, or well-being. Less is known about how the institutional privileging of algorithmically visible metrics relates to professionals’ perceptions of their relative worth within competence hierarchies, even when employment is not immediately at risk.

When AI-generated or digitally benchmarked metrics become the most visible and consequential evaluative referents, professionals may infer that less algorithmically legible contributions—mentoring, contextual expertise, collegial judgment, and pedagogical depth—are comparatively under-recognized (Chun et al., 2024). Such inferences reflect a status-based appraisal of whether broader contribution is fairly valued under the prevailing evaluation regime. This appraisal is termed algorithmic relative devaluation (ARD)—the perception that professional contribution is comparatively downgraded when AI-mediated or digitally benchmarked standards become institutionally privileged evaluative referents. ARD differs analytically from job insecurity, technostress, perceived unfairness, and professional identity threat; it captures a specific inference about comparative recognition within a changing evaluative order rather than a generalized fear of displacement or procedural violation (Petriglieri, 2011; Selenko et al., 2022).

Three evaluation design features are theorized as antecedents of ARD: algorithmic visibility, benchmark intensity, and algorithmic explainability. Algorithmic visibility refers to the salience of algorithmic or metric-based indicators within evaluation routines. Benchmark intensity refers to the degree of continuous comparison against metricized thresholds. Algorithmic explainability refers to the perceived intelligibility and reviewability of the evaluation logic. Visibility and benchmark intensity capture the prominence and force of algorithmic evaluation, whereas explainability captures whether evaluations can be understood and meaningfully reviewed (Huo et al., 2025; Jian et al., 2000). These features appear across AI-powered systems and algorithmically mediated tools such as citation rankings, productivity dashboards, or grant-scoring systems (Schmidt et al., 2025). The argument is not that AI is uniquely devaluing, but that the institutional logic shared across these systems—making selected indicators visible, comparable, and authoritative—may shape how professionals perceive their relative contribution. This distinction is important because AI-enabled and metric-based evaluation should not be conflated: both can operate through a common evaluative mechanism, but they differ in opacity, scale, and technological infrastructure (Bankins and Formosa, 2023; Newlands, 2021).

Within this framework, ARD is expected to channel evaluation experiences into two distinct forms of career response. Adaptive career reconfiguration denotes constructive efforts to realign professional value with changing evaluation demands, such as skill development, digital tool integration, and expertise repositioning. Defensive career reconfiguration denotes protective efforts to distance from metric-intensive or algorithmically exposed domains, for example by avoiding certain roles, shifting emphasis to less visible work, or reframing contribution away from algorithmically legible outputs (Callari and Lohse, 2025; Li and Li, 2025). Prior studies show that AI-related threat and dominance perceptions can be associated with both approach-oriented responses, such as career exploration, adaptability, and job crafting, and avoidance responses, such as withdrawal or defensive job crafting (Kong et al., 2023b; Presbitero and Teng-Calleja, 2022). These responses may carry different implications for perceived career sustainability, understood in terms of the continuity, adaptability, and meaningfulness of one’s career trajectory over time (De Vos et al., 2020). Responsible AI governance—encompassing transparency, fairness safeguards, appeal procedures, participation, and procedural accountability—is considered as a boundary condition that may buffer or amplify these dynamics by shaping the perceived legitimacy and contestability of algorithmic evaluation (Kumari and Tiwari, 2026).

The empirical context is Chinese higher education, selected as a theoretically stringent and revealing setting rather than as a basis for broad generalization. Chinese academic evaluation is heavily metricized, integrating citation dashboards, journal tier rankings, grant-tracking platforms, AI-assisted teaching analytics, and research productivity benchmarks into formal promotion and appraisal systems (Mai, 2025). Policy documents associated with the “Double First-Class” initiative specify numerical targets for publications, journal categories, and external funding, and promotion criteria in many universities explicitly tie advancement to quantified benchmarks (Liu, 2021; Marginson, 2022). This concentration of algorithmically visible and digitally benchmarked evaluation makes it possible to examine how faculty members perceive and respond to the institutional privileging of computational standards. At the same time, institutional and cultural factors—including centralized academic governance, norms around institutional authority, and the specific architecture of the “Double First-Class” evaluation regime—are likely to shape how ARD and its consequences manifest. These boundary conditions are treated as explicit limitations rather than as grounds for unqualified generalization to all AI-mediated professional contexts.

Using a three-wave time-lagged survey design analyzed through PLS-SEM with *N* = 448 Chinese faculty members, the analysis addresses three research questions:

1. How are algorithmic visibility, benchmark intensity, and algorithmic explainability associated with perceptions of algorithmic relative devaluation?
2. How is algorithmic relative devaluation associated with adaptive and defensive career reconfiguration, and how are these responses related to perceived career sustainability?
3. How does responsible AI governance condition these relationships?

The contribution is threefold. First, ARD is positioned as a contextual refinement of existing AI-threat, job insecurity, status, and identity-based perspectives by specifying a comparative recognition appraisal under algorithmically privileged evaluation standards. Second, the study examines how ARD is associated with both adaptive and defensive career reconfiguration, which may carry different implications for perceived career sustainability. Third, it theorizes responsible AI governance as an ambivalent boundary condition that may buffer perceived devaluation when it broadens contestability, but may intensify devaluation when it legitimizes narrow benchmarking. Taken together, the study reframes AI-enabled and digitally benchmarked evaluation not merely as productivity infrastructure or a source of stress, but as a context in which professionals may reassess the relative recognition of their contribution within metricized institutional environments.

## 2 Literature review

### 2.1 Algorithmic evaluation and career dynamics

Research on AI and work has increasingly moved beyond whether AI replaces human labour to examine how AI-mediated systems shape evaluation, control, and career experience. In organizational settings, algorithmic systems classify performance, generate comparative indicators, support managerial decisions, and standardize assessments across individuals or units (Bankins et al., 2022; Engstrom and Haim, 2023). These systems matter because evaluation is not only a technical act of measurement; it is also an institutional process through which competence, contribution, and professional worth are recognized. When digital indicators are embedded in promotion, performance review, workload allocation, or funding decisions, they influence how professionals interpret their standing within organizational hierarchies.

It is therefore important to distinguish AI-enabled evaluation from broader algorithmic and metric-based evaluation. AI-enabled evaluation refers to assessment systems that use machine learning, predictive analytics, or AI-assisted judgment to support evaluative decisions. Algorithmic evaluation is broader and includes rule-based or computational systems that translate performance into scores, rankings, or decision categories. Metric-based evaluation is broader still, encompassing bibliometric indicators, dashboards, journal classifications, citation counts, and productivity indices that may or may not involve AI. These systems are not identical, but they share an evaluative logic: they privilege indicators that can be rendered visible, comparable, and actionable. In metric-intensive settings such as higher education, this logic is consequential because professional contribution often includes qualitative, relational, and long-term forms of work that are difficult to capture through standardized indicators.

Existing scholarship has documented several consequences of AI and algorithmic evaluation. Studies on algorithmic management show that digital systems can intensify monitoring, redistribute discretion, and structure managerial control through data-based performance indicators (Williams, 2025). Research on AI-enabled decision support shows that organizations increasingly rely on computational tools to classify performance, predict outcomes, and support evaluation in knowledge-intensive environments (Alam et al., 2025b; Chuang et al., 2024). Related work links AI-mediated monitoring to technostress, emotional exhaustion, autonomy loss, and job insecurity, particularly where digital oversight is continuous or opaque (Yılmaz, 2025). Career-focused studies emphasize adaptive responses to technological change, including upskilling, reskilling, learning orientation, job crafting, and employee–AI collaboration as pathways to employability or career sustainability (Ahmad and Bilal, 2024; Ersanlı et al., 2025; Rastogi et al., 2025).

These streams establish that AI-mediated evaluation affects work experience and career behavior, but they leave underdeveloped a more specific question: how do algorithmic and digitally benchmarked evaluation systems affect professionals’ perceptions of their relative value within institutional evaluation regimes? Existing explanations focus on employment threat, psychological strain, surveillance, trust, fairness, or skill adaptation. These mechanisms are important, yet they do not fully capture situations where professionals are not primarily afraid of losing their jobs, but perceive that the basis for recognizing their contribution has shifted toward algorithmically visible indicators. In such cases, the central issue is not displacement or stress alone, but comparative recognition—whether one’s broader contribution continues to be valued when institutional evaluation privileges what can be measured, ranked, predicted, or displayed.

This gap is especially salient in Chinese higher education. Academic evaluation in this context is shaped by citation dashboards, journal classifications, grant indicators, teaching analytics, and digitally benchmarked performance systems (Cheng, 2023; Mai, 2025). Some of these systems are AI-enabled; others are algorithmic or bibliometric. Their common effect is not simply to automate academic judgment, but to render selected indicators more visible and institutionally consequential. Faculty may therefore experience evaluation not only as monitoring or performance pressure, but as a narrowing of the standards through which academic contribution is recognized. This makes Chinese higher education a stringent context for examining how algorithmic and digitally benchmarked evaluation may be associated with perceived relative devaluation.

Against this backdrop, the present study positions algorithmic relative devaluation (ARD) as a mechanism linking evaluation design to career dynamics. ARD refers to professionals’ perception that their broader contribution is comparatively downgraded when algorithmic or digitally benchmarked standards become privileged evaluative referents. This mechanism extends AI–work research by shifting attention from whether AI threatens jobs or induces strain to how algorithmically visible standards may reshape perceived recognition within an evaluative order. It also extends career sustainability research by explaining why professionals may engage in adaptive or defensive career reconfiguration even in the absence of immediate employment threat (De Vos et al., 2020).

Table 1 summarizes the strands of literature that inform this positioning. Prior research has examined AI capability, algorithmic control, monitoring-related strain, AI trust, job crafting, and career adaptation. However, it has not specified the comparative worth inference through which algorithmic evaluation design may become linked to perceived professional standing and sustainable career responses. This omission provides the basis for the present model, which examines whether perceived algorithmic evaluation features are associated with ARD and how ARD relates to career reconfiguration and perceived career sustainability in a metric-intensive academic context.

**Table 1:** Literature matrix.

Source	Context	Core focus	Method	Key findings relevant to ARD positioning
Huo et al. (2025)	AI-enabled workplaces	AI explainability & job crafting	Multi-wave survey	Explainability reduces AI threat perceptions; shapes approach vs. avoidance job crafting
Bankins et al. (2022)	Algorithmic management environments	Algorithmic control & legitimacy	Conceptual + empirical	Algorithmic systems function as governance mechanisms reshaping authority and legitimacy
Kong et al. (2023b)	AI-augmented organizations	AI trust & career sustainability	Survey-based empirical study	AI trust → employee–AI collaboration → career sustainability
Low et al. (2025)	Technology adoption contexts	Effort expectancy & performance expectancy	UTAUT-based survey	AI expectations influence engagement and performance perceptions
Hamouche et al. (2023)	AI transformation workplaces	AI-induced stress & psychological outcomes	Systematic review	AI implementation linked to technostress and emotional strain
Magni et al. (2023)	AI-assisted evaluation settings	Creativity evaluation & effort perception	Experimental study	AI-based evaluations alter perceived fairness and effort recognition
Ateeq et al. (2025)	AI-driven leadership systems	AI & employee engagement	Empirical analysis	AI systems reshape engagement dynamics through leadership mediation
This study	AI-enabled and digitally benchmarked professional evaluation in Chinese higher education	Algorithmic evaluation features, perceived relative devaluation, and career sustainability	Three-wave time-lagged survey; PLS-SEM	Examines whether perceived visibility, benchmark intensity, and explainability are associated with ARD, and whether ARD relates to adaptive/defensive career reconfiguration and perceived career sustainability under responsible AI governance

### 2.2 Theoretical underpinning

The problem examined in this study is not simply that AI-enabled evaluation introduces new tools into professional assessment, but that algorithmic and digitally benchmarked systems may alter the terms under which professional worth becomes institutionally recognizable. Existing AI–work research has focused on automation risk, job insecurity, technostress, surveillance, and fairness, explaining how AI threatens employment or intensifies monitoring, but saying less about a subtler status problem: professionals may remain employed and competent while perceiving that the evaluative basis for recognition has narrowed toward algorithmically visible indicators.

Algorithmic relative devaluation (ARD) captures this problem. ARD refers to professionals’ perception that their broader contribution is comparatively downgraded when algorithmic or digitally benchmarked standards become privileged evaluative referents. Its focus is not generalized AI anxiety or fear of job loss, but relative valuation: the sense that institutions increasingly recognize what can be measured, ranked, and displayed, while less measurable forms of contribution become comparatively less visible.

The theoretical logic integrates status processes research and professional identity theory. Status research shows that hierarchies are sustained through signals that become socially recognized as evidence of competence and worth (Berger et al., 1972; Piazza and Castellucci, 2014; Ridgeway, 2014). Professional identity theory emphasizes that recognition matters for how individuals understand their role, value, and career continuity (Ashforth and Mael, 1989; Petriglieri, 2011). ARD sits at the intersection of these logics: it is a status appraisal that becomes career-relevant because professional identity depends partly on whether one’s contribution is institutionally recognized.

Commensuration research sharpens this status logic. Quantified indicators do not merely represent work; they transform heterogeneous contributions into comparable units and reorganize attention around what can be counted (Espeland and Sauder, 2007; Espeland and Stevens, 1998). In metricized regimes, valuable work such as mentorship, pedagogical judgment, collegial service, and intellectual risk-taking may be partially commensurable at best. When evaluation systems privilege what can be counted, displayed, and benchmarked, these less measurable contributions risk losing relative recognition even if they remain essential to professional practice.

Chinese higher education provides a stringent context for examining this process because academic careers are embedded in metric-intensive regimes that include citation dashboards, journal classifications, grant indicators, teaching analytics, and digitally benchmarked performance systems (Mai, 2025). Some of these systems are AI-enabled, others algorithmic, and others bibliometric. Their shared logic is the privileging of indicators that are visible, comparable, and actionable. ARD is therefore theorized not as a reaction to “AI” as technology per se, but as a response to the institutional elevation of algorithmically legible standards as evidence of professional worth.

The internal logic of ARD follows from this recognition problem. The comparison is not “human versus AI” but broader professional contribution versus algorithmically visible indicators; the source of devaluation is the evaluation regime that grants authority to such indicators; and the devalued object is work that loses recognition when institutional attention shifts toward measurable outputs. The mechanism is comparative worth inference: professionals infer that what they contribute is valued less relative to what the system can measure, rank, and display.

ARD overlaps with, but is not reducible to, adjacent constructs. Job insecurity concerns employment loss; ARD concerns whether one’s contribution remains valued within an evaluative order. AI threat captures generalized apprehension toward AI; ARD concerns comparative downgrading under algorithmically privileged standards. Perceived unfairness focuses on procedural or distributive justice (Colquitt, 2001); ARD focuses on the scope of recognition. Professional identity threat concerns harm to role-based self-definition (Petriglieri, 2011); ARD precedes such threat by indicating that institutional referents no longer fully recognize one’s contribution. Status threat concerns possible loss of standing; ARD specifies the source of that loss as the privileging of algorithmically visible criteria. ARD is thus best understood as a context-specific extension of status, identity, and justice research into algorithmically mediated evaluation regimes.

The model’s evaluation features represent different routes through which this status appraisal may emerge. Algorithmic visibility concerns the salience of machine-readable indicators—the extent to which professionals see their work represented through algorithmic outputs. Benchmark intensity concerns the force and frequency of comparison—the extent to which evaluation is experienced as continuous alignment with quantitative thresholds. Algorithmic explainability is structurally different: it concerns whether professionals can understand, interpret, and contest the evaluative logic behind algorithmic judgments (Jian et al., 2000; Lee and See, 2004). Visibility and benchmark intensity are therefore expected to heighten ARD by strengthening the perceived authority of algorithmically visible standards, whereas explainability may reduce ARD when it makes evaluation procedurally intelligible and preserves space for professional judgment (Huo et al., 2025; Zhang and Tang, 2024).

Professional identity theory explains why ARD becomes career-relevant. Professionals respond not only to material outcomes but also to whether institutions recognize the forms of contribution that sustain their role identity (Ashforth and Mael, 1989; Petriglieri, 2011). When evaluation increasingly privileges algorithmically legible outputs, professionals may experience a mismatch between what they regard as valuable work and what the institution appears to value. ARD captures this mismatch at the level of evaluative recognition before it necessarily escalates into broader identity threat.

This mismatch may prompt career reconfiguration, but such reconfiguration is not uniformly adaptive. Adaptive reconfiguration involves efforts to regain recognition within the changing evaluation regime (e.g., acquiring AI-related competencies, integrating digital tools), whereas defensive reconfiguration involves efforts to protect valued work from narrow benchmarking (e.g., distancing from metric-intensive roles or emphasizing less exposed domains). Their implications for career sustainability may differ, as adaptive strategies can enhance perceived viability while deepening engagement with metric regimes, whereas defensive strategies can preserve identity at the cost of alignment with emerging expectations (Ashforth and Mael, 1989).

Responsible AI governance is theorized with similar ambivalence. From an organizational justice perspective, transparency, fairness safeguards, appeal procedures, and participation can reduce ARD by making evaluation more procedurally legitimate and preserving channels through which professionals can contextualize algorithmic outputs (Colquitt, 2001). From an institutional legitimacy perspective, the same governance can normalize and legitimize intensive benchmarking by making it appear responsible and objective, even when the underlying definition of valued contribution remains narrow (Newlands, 2021; Suchman, 1995). Governance may therefore buffer ARD when it broadens recognition, but amplify ARD when it legitimizes narrow, algorithmically visible criteria.

The framework thus avoids treating algorithmic evaluation as inherently harmful or inherently efficient. Its central claim is narrower: algorithmic and digitally benchmarked systems become career-relevant when they alter the perceived terms of professional recognition. The model examines whether perceived evaluation features are associated with ARD, and whether this appraisal relates to career reconfiguration and perceived career sustainability in a highly metricized academic context.

**Figure 1:**
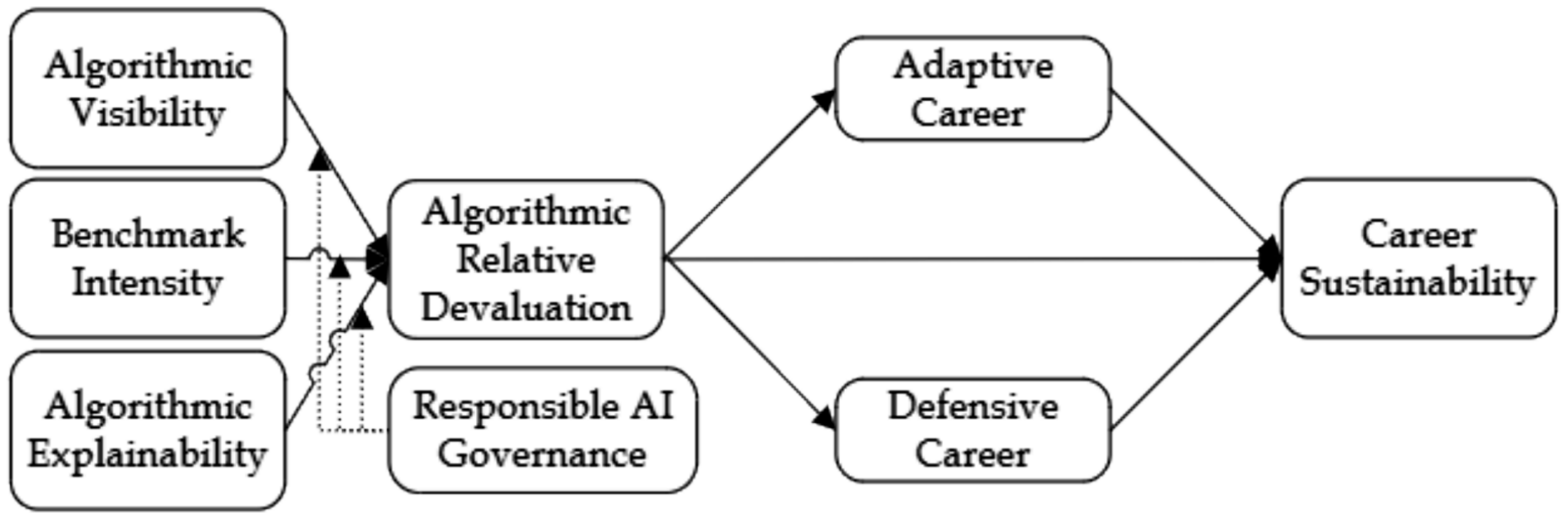
Research framework.

Figure 1 summarizes this framework. The model examines associations between perceived algorithmic evaluation features, ARD, career reconfiguration, and perceived career sustainability in a highly metricized academic context, without claiming that AI objectively restructures professional hierarchies.

### 2.3 Hypothesis development

Algorithmic and digitally benchmarked evaluation systems do not only make performance measurable; they also shape which forms of professional contribution become visible, comparable, and institutionally consequential. Building on the preceding theorization, algorithmic relative devaluation (ARD) refers to professionals’ perception that their broader contribution is comparatively downgraded when algorithmically visible standards become privileged evaluative referents. ARD focuses on relative valuation within an evaluation regime rather than employment continuity, generalized AI threat, or procedural fairness; it captures the perception that one’s contribution is valued less when institutional recognition moves toward algorithmically measurable indicators.

#### 2.3.1 Algorithmic evaluation features and ARD

Algorithmic visibility should heighten ARD because visible dashboards, rankings, and digital scores make machine-readable outputs salient as markers of professional worth. Visibility is not neutral disclosure; it directs institutional attention toward what can be counted and compared. When algorithmic outputs become prominent in evaluation routines, professionals may infer that less visible forms of contribution (e.g., mentoring, pedagogical judgment, collegial service) receive weaker recognition, consistent with evidence that algorithmic evaluation can feel disrespectful when it fails to account for individualized contribution (Chun et al., 2024). Thus:

*H1a*: Algorithmic visibility is positively associated with algorithmic relative devaluation.

Benchmark intensity should also be associated with higher ARD because it can transform metrics from informational indicators into continuous evaluative pressure. Whereas visibility makes algorithmic indicators salient, benchmark intensity makes them consequential by repeatedly positioning professionals against thresholds, rankings, or productivity norms. Such comparison narrows the perceived basis of professional worth and strengthens the authority of quantified indicators. Prior research links intensive algorithmic control to autonomy loss, alienation, and defensive responses (Keegan and Meijerink, 2025; Liu et al., 2024; Ofem, 2025; Teng et al., 2023). Thus:

*H1b*: Benchmark intensity is positively associated with algorithmic relative devaluation.

Algorithmic explainability is structurally different from visibility and benchmark intensity. It does not simply make evaluation more visible; it concerns whether professionals can understand, interpret, and contest the logic behind algorithmic judgments. Explainability should reduce ARD when it makes evaluation criteria procedurally intelligible and preserves space for professional judgment, thereby weakening the perception that algorithmic standards operate as opaque authorities (Huo et al., 2025). At the same time, explainability is not inherently protective: explanations that merely rationalize narrow benchmarks may legitimize evaluative narrowing (Bauer et al., 2023; Muhammad et al., 2025). Nevertheless, the baseline expectation is devaluation-reducing. Thus:

*H1c*: Algorithmic explainability is negatively associated with algorithmic relative devaluation.

#### 2.3.2 ARD, career reconfiguration, and career sustainability

ARD should prompt career reconfiguration because perceived comparative devaluation signals that existing forms of contribution may no longer secure institutional recognition. However, reconfiguration is not a homogeneous response. Adaptive reconfiguration reflects efforts to regain recognition within the changing evaluation regime by developing new competencies, integrating AI-related tools, or repositioning expertise. Prior work shows that AI-related uncertainty and perceived AI dominance can stimulate career exploration, adaptability, learning, and job crafting (Burhan, 2024; Kong et al., 2023b; Presbitero and Teng-Calleja, 2022). Thus:

*H2a*: Algorithmic relative devaluation is positively associated with adaptive career reconfiguration.

Defensive reconfiguration reflects efforts to protect valued work from perceived misrecognition, such as distancing from metric-intensive roles, avoiding AI-exposed tasks, or emphasizing domains less subject to algorithmic comparison. ARD can plausibly generate such responses because it signals not only a need to adapt, but also a perceived narrowing of what the institution values. Evidence on avoidance-oriented job crafting, knowledge hiding, withdrawal, and rumination under algorithmic control supports this defensive pathway (Li and Li, 2025; L. Liu and Liu, 2024; Teng et al., 2023). Thus:

*H2b*: Algorithmic relative devaluation is positively associated with defensive career reconfiguration.

ARD should also weaken perceived career sustainability. Career sustainability concerns the perceived continuity, adaptability, and long-term viability of one’s career trajectory (De Vos et al., 2020). When professionals perceive that their broader contribution is losing recognition under algorithmically privileged standards, they may become less confident that their career remains meaningful and viable in the prevailing evaluation regime (Selenko et al., 2022). Thus:

*H2c*: Algorithmic relative devaluation is negatively associated with career sustainability.

Adaptive reconfiguration should enhance perceived career sustainability because it renews fit between professional value and changing evaluative expectations. By developing new competencies, integrating digital tools, and repositioning expertise, professionals may strengthen perceived adaptability and future viability. Prior research links AI knowledge, employee–AI collaboration, job crafting, and learning orientation to career sustainability and person–job fit (Duong et al., 2026; Kong et al., 2023a; Selenko et al., 2022). Thus:

*H3a*: Adaptive career reconfiguration is positively associated with career sustainability.

Defensive reconfiguration may have the opposite implication. Although it may protect valued identity claims in the short term, distancing from AI-exposed or metric-intensive domains can reduce perceived adaptability and alignment with emerging institutional expectations, echoing findings that avoidance responses and withdrawal behaviors undermine longer-term employability and career continuity (Rastogi et al., 2025; Selenko et al., 2022). Thus:

*H3b*: Defensive career reconfiguration is negatively associated with career sustainability.

#### 2.3.3 Moderating role of responsible AI governance

Responsible AI governance conditions whether algorithmic evaluation is interpreted as legitimate support or narrowed recognition. Governance practices such as transparency, appeal procedures, fairness safeguards, participation, and ethical oversight can reduce ARD by making evaluation more accountable and preserving channels for contextualizing algorithmic outputs (Blader and Tyler, 2003; Colquitt, 2001). Yet governance is not uniformly protective: when it formalizes intensive benchmarking without broadening what counts as valued contribution, it may legitimize rather than weaken algorithmic authority (Suchman, 1995; Newlands, 2021).

This dual role implies differentiated moderation. Governance can channel ARD toward adaptive rather than defensive responses when professionals perceive evaluation as fair and negotiable, consistent with evidence that ethical climates and responsible leadership foster constructive engagement with AI-induced change (Shin, 2021; Kim and Lee, 2026). Governance may also attenuate the visibility–ARD relationship by making visible indicators more accountable and open to challenge. Conversely, governance can strengthen the benchmark intensity–ARD relationship when formal safeguards make intensive benchmarking appear institutionally endorsed, and can amplify the devaluation-reducing role of explainability when explanations are embedded in fair procedures (Bauer et al., 2023; Xiao et al., 2023). Accordingly:

*H4a*: Responsible AI governance weakens the positive relationship between algorithmic visibility and algorithmic relative devaluation.

*H4b*: Responsible AI governance strengthens the positive relationship between benchmark intensity and algorithmic relative devaluation.

*H4c*: Responsible AI governance strengthens the negative relationship between algorithmic explainability and algorithmic relative devaluation.

#### 2.3.4 Indirect effects via ARD

ARD is theorized as the key appraisal through which algorithmic evaluation features become linked to career reconfiguration. Unlike broader mediators such as job insecurity, generic AI threat, or technostress (Hamouche et al., 2023; Presbitero and Teng-Calleja, 2022), ARD focuses on comparative recognition—the perception that one’s contribution is being downgraded relative to algorithmically visible standards. Algorithmic visibility and benchmark intensity should heighten this appraisal by making machine-readable indicators more salient and consequential, whereas explainability should weaken it by rendering evaluation more intelligible and reviewable (Huo et al., 2025).

Prior research shows that AI-related perceptions influence career exploration, job crafting, and adaptability through threat- and insecurity-related appraisals (Burhan, 2024; Li and Li, 2025). ARD refines this mechanism by specifying that what is appraised is not only future employment risk, but current relative value under algorithmically privileged metrics. In response to perceived comparative devaluation, some professionals may pursue adaptive reconfiguration (e.g., skill development, AI collaboration), while others may engage in defensive reconfiguration (e.g., distancing from metric-intensive roles), consistent with evidence on both approach and avoidance responses to AI and algorithmic control (Kong et al., 2023b; Liu et al., 2024; Teng et al., 2023). Within this constraint, ARD is posited as the most proximal comparative-worth mechanism linking perceived evaluation design to adaptive and defensive career reconfiguration. Accordingly:

*H5a*: Algorithmic relative devaluation mediates the relationship between algorithmic visibility and adaptive career reconfiguration.

*H5b*: Algorithmic relative devaluation mediates the relationship between benchmark intensity and adaptive career reconfiguration.

*H5c*: Algorithmic relative devaluation mediates the relationship between algorithmic explainability and adaptive career reconfiguration.

*H5d*: Algorithmic relative devaluation mediates the relationship between algorithmic visibility and defensive career reconfiguration.

*H5e*: Algorithmic relative devaluation mediates the relationship between benchmark intensity and defensive career reconfiguration.

*H5f*: Algorithmic relative devaluation mediates the relationship between algorithmic explainability and defensive career reconfiguration.

## 3 Methodology

### 3.1 Research design, context, and sample

A three-wave time-lagged survey design was used to examine the moderated mediation framework and reduce consistency bias/common method inflation through temporal separation (Hamouche et al., 2023). At T1, respondents reported algorithmic visibility, benchmark intensity, algorithmic explainability, responsible AI governance, exposure to algorithmic or digitally benchmarked evaluation systems. At T2, 2 weeks later, they reported algorithmic relative devaluation (ARD). At T3, after another 2 weeks, they reported adaptive career reconfiguration, defensive career reconfiguration, and career sustainability. The two-week lag allowed appraisal-based responses to emerge while limiting attrition, consistent with AI–career panel research (Presbitero and Teng-Calleja, 2022). The design supports temporal ordering but not causal inference; findings are therefore interpreted as theoretically ordered associations.

China’s higher education sector was selected because academic evaluation is strongly shaped by citation dashboards, journal classifications, grant-monitoring platforms, teaching analytics, and AI-assisted evaluation tools (Mai, 2025). Although these systems vary in whether they are AI-enabled, algorithmic, or bibliometric, they share a common evaluative logic: making selected indicators visible, comparable, and consequential for promotion, resources, and recognition. This makes the context appropriate for examining perceived relative devaluation under digitally benchmarked standards.

The target population comprised full-time faculty members with at least 1 year of academic experience and confirmed exposure to AI-assisted, algorithmic, or digitally benchmarked evaluation systems. Exposure was verified through screening items covering citation dashboards, journal-ranking systems, AI-assisted teaching analytics, grant-monitoring platforms, algorithmic workload allocation, productivity dashboards, and similar tools. Because respondents could report multiple forms of exposure, exposure intensity was calculated as the number of systems reported and used in robustness analyses. The distribution of exposure types is reported in Appendix Table T6.

Participants were recruited from “Double First-Class” universities, provincial public universities, and applied or teaching-oriented institutions across Eastern, Central, and Western China. Academic rank followed the Chinese higher education system. “Lecturer” denotes an early-career academic rank broadly comparable to an assistant-professor-level or junior faculty position, although equivalence varies by institution. “Associate professor” and “Professor” denote senior ranks, while “Teaching/Research Fellow” refers to non-tenured or fixed-term teaching/research positions. This clarification is relevant because rank may shape promotion pressure, benchmark exposure, and vulnerability to metricized evaluation.

Following ethics approval, data were collected between November 2025 and January 2026 through department-level emails, faculty-only WeChat academic groups, alumni networks, and snowball referrals. The Wenjuanxing survey was voluntary and anonymous, and cross-wave matching used self-generated identification codes. Valid responses were T1 = 712, T2 = 563, and T3 = 448, yielding a final matched sample of 448 and an overall retention rate of 62.9%. Attrition analyses comparing completers and dropouts on demographics, exposure types, and T1 focal variables showed no significant differences in the main predictors at the 0.05 level. Detailed attrition results are reported in Appendix Table T4. The final sample exceeds recommended thresholds for PLS-SEM models with mediation and moderation (Hair et al., 2022) and satisfies *a priori* power requirements for medium effects, power = 0.95, *f*^2^ = 0.15, and *α* = 0.05 (Cohen, 1988).

### 3.2 Measures and data quality

All constructs were measured using multi-item reflective scales adapted to the context of AI-enabled and digitally benchmarked academic evaluation. The items referred to citation dashboards, AI-assisted evaluation tools, teaching analytics, grant-monitoring systems, journal-ranking indicators, and related digital evaluation practices. All scales used a five-point Likert format ranging from 1 = strongly disagree to 5 = strongly agree. Full item wording and sources are reported in Appendix Table T1.

Algorithmic visibility, benchmark intensity, and algorithmic explainability were adapted from algorithmic management, work-stressor, and trust-in-automation research. These constructs capture, respectively, the salience of algorithmic or metric-based indicators in evaluation routines, the perceived pressure and frequency of comparison against metricized benchmarks, and the perceived intelligibility and reviewability of evaluation logic (Jian et al., 2000; Lee and See, 2004). Algorithmic relative devaluation (ARD) captures perceived comparative downgrading of professional contribution under algorithmically privileged standards. The ARD items were explicitly worded to reflect relative valuation rather than general AI threat, job insecurity, perceived unfairness, or identity threat.

Career reconfiguration was modeled as two separate constructs. Adaptive career reconfiguration captures skill development, digital tool integration, and expertise repositioning. Defensive career reconfiguration captures distancing from metric-intensive domains, avoidance of AI-exposed roles, and emphasis on forms of contribution that are less visible to algorithmic evaluation systems. Career sustainability follows De Vos et al. (2020), while responsible AI governance reflects transparency, fairness safeguards, appeal procedures, participation, and procedural accountability (Colquitt, 2001; Blader and Tyler, 2003).

All constructs were coded so that higher scores represent higher levels of the named construct. Higher scores for career sustainability indicate stronger perceived career sustainability, and higher scores for algorithmic explainability indicate greater perceived intelligibility and reviewability of algorithmic evaluation.

The instrument was refined through a cognitive pretest (*n* = 20) and a pilot study (*n* = 88). Data-quality controls included IP restriction, browser cookies, anonymized matching codes, instructional manipulation checks, response-time screening, and long-string analysis (Meade and Craig, 2012). Cases failing these checks were removed before final analysis.

### 3.3 Analytical procedures

PLS-SEM was used in SmartPLS to estimate a model involving multiple mediation paths, moderation effects, non-normal survey data, and explained variance in ARD and career-related outcomes (Hair et al., 2022). Measurement quality was evaluated using indicator loadings, Cronbach’s alpha, composite reliability, average variance extracted, HTMT ratios, and item-level diagnostics. Given the conceptual proximity of ARD to related perceptions, discriminant validity was additionally assessed through CB-SEM model comparisons between the proposed multi-factor structure and more restrictive alternatives. Appendix Table T2 reports global fit indices for the proposed eight-factor model, one-factor model, and theoretically relevant construct-combined models.

Structural paths were estimated with 10,000 bootstrap resamples, reporting standardized coefficients, t-values, *p*-values, 95% confidence intervals, effect sizes, and R^2^ values for endogenous constructs. Adaptive and defensive career reconfiguration were retained as separate endogenous variables throughout the analysis. Predictive relevance was examined using *Q*^2^ and PLSpredict (Alam et al., 2025a). Because the linear benchmark model performed comparably or slightly better for some indicators, predictive results are interpreted cautiously. Accordingly, the model is positioned as explanatory and theory-driven rather than as demonstrating superior out-of-sample prediction.

Descriptive statistics and bivariate correlations among focal constructs are reported in Appendix Table T3 to improve transparency and allow assessment of construct overlap. Robustness checks compared the baseline model with alternative specifications, including a marker-adjusted model, and a Gaussian Copula specification.

### 3.4 Robustness checks

Several robustness checks were conducted to assess the stability of the findings. Common method bias was addressed procedurally through temporal separation, anonymity, item separation across survey waves, neutral wording, and data-quality screening. It was also assessed statistically using a theoretically unrelated marker variable and full collinearity VIFs (Kock, 2015; Lindell and Whitney, 2001). The marker-adjusted model was estimated as a method-control specification to examine whether focal path directions and significance remained stable after accounting for potential common method variance.

The R^2^ values from the marker-adjusted model are reported for transparency. However, because the marker-adjusted specification includes an additional method-control component, these R^2^ values are not interpreted as direct improvements over the baseline model. Instead, the marker-adjusted model is used to assess the stability of the substantive path pattern after controlling for potential method-related variance.

Potential endogeneity was assessed using the Gaussian Copula approach (Park and Gupta, 2012). The Gaussian Copula model was estimated separately from the baseline model by including copula terms for the relevant endogenous predictors. The purpose of this test was to examine whether the direction and significance of the focal paths remained stable after accounting for potential non-normal endogeneity.

Table 2 compares the baseline, marker-adjusted, and Gaussian Copula specifications. The core structural pattern remains stable across the three specifications. In particular, the paths from algorithmic visibility and algorithmic explainability to ARD, from ARD to adaptive and defensive career reconfiguration, and from adaptive and defensive career reconfiguration to career sustainability remain significant. The benchmark intensity to ARD path and the direct ARD to career sustainability path also remain significant, although their smaller t-values suggest that these relationships should be interpreted more cautiously than the stronger ARD-to-career-reconfiguration paths.

**Table 2:** Robustness checks across baseline, marker-adjusted, and Gaussian copula models.

Path/endogenous Construct	Baseline *t*	Baseline *p*	Marker-adjusted *t*	Marker-adjusted *p*	Gaussian Copula *t*	Gaussian Copula *p*
AC → CS	11.284	<0.001	9.399	<0.001	10.436	<0.001
AE → ARD	6.543	<0.001	6.211	<0.001	5.284	<0.001
ARD → AC	17.675	<0.001	5.311	<0.001	16.972	<0.001
ARD → CS	3.962	<0.001	2.965	0.002	2.846	0.002
ARD → DC	24.011	<0.001	9.072	<0.001	21.364	<0.001
AV → ARD	7.779	<0.001	7.660	<0.001	6.937	<0.001
BI → ARD	3.130	0.001	2.706	0.003	2.521	0.006
DC → CS	3.057	0.001	2.353	0.009	2.674	0.004
R^2^: AC	0.353	—	0.548	—	0.358	—
R^2^: ARD	0.679	—	0.679	—	0.682	—
R^2^: CS	0.666	—	0.677	—	0.670	—
R^2^: DC	0.491	—	0.607	—	0.497	—

The table reports *t*-values and *p*-values only because standardized coefficients are reported in the main structural model. *R*^2^ values are reported for endogenous constructs.

Additional robustness checks are reported in the Appendix to avoid diluting the main theoretical model. Appendix Table T4 reports attrition analyses comparing completers and dropouts. Appendix Table T5 reports exposure heterogeneity robustness by including exposure intensity as an additional control. Together, these checks address potential concerns about panel attrition, heterogeneous exposure to AI-assisted or digitally benchmarked evaluation systems.

## 4 Data analysis

Table 3 presents the demographic profile of the final matched sample (N = 448). The sample is moderately male-skewed (55.4%), with representation across age groups. Most respondents were between 31 and 50 years old (71.0%), representing the core mid-career academic cohort likely to be actively engaged in promotion, grant competition, and metric-based evaluation processes.

**Table 3:** Demographics of the respondents.

Variable	Category	*n*	%
Gender	Male	248	55.4
	Female	198	44.2
	Prefer not to say	2	0.4
Age (years)	25–30	54	12.1
	31–40	172	38.4
	41–50	146	32.6
	51–60	76	17.0
Academic rank	Lecturer	118	26.3
	Associate professor	181	40.4
	Professor	97	21.7
	Teaching/research fellow (non-tenured track)	52	11.6
Years of academic experience	1–5 years	72	16.1
	6–10 years	134	29.9
	11–20 years	167	37.3
	21 + years	75	16.7
Institution type	Double First-Class (research-intensive)	164	36.6
	Provincial Public University	193	43.1
	Applied/Teaching-Oriented University	91	20.3
Disciplinary field	Engineering & technology	121	27.0
	Natural sciences	84	18.8
	Social sciences	103	23.0
	Business & management	79	17.6
	Humanities & arts	61	13.6
Administrative role	No administrative role	312	69.6
	Department-level role	98	21.9
	School/faculty-level leadership	38	8.5

Associate professors constituted the largest group (40.4%), followed by lecturers (26.3%) and full professors (21.7%). This distribution is analytically appropriate because associate professors in China are often subject to strong promotion pressure under citation- and grant-based benchmarking systems. Years of academic experience were broadly distributed, with 83.9% of respondents reporting at least 6 years of academic experience, indicating substantial familiarity with institutional evaluation regimes.

Institutionally, respondents were drawn from research-intensive “Double First-Class” universities (36.6%), provincial public universities (43.1%), and applied institutions (20.3%), supporting cross-institutional heterogeneity while maintaining exposure to nationally standardized performance evaluation systems. Disciplinary representation was diverse, with engineering and technology (27.0%) and social sciences (23.0%) forming the largest segments.

Most respondents (69.6%) did not hold administrative roles, suggesting that the perceptions captured primarily reflect faculty-level rather than managerial interpretations of AI-based evaluation. Overall, the demographic profile supports the study’s contextual alignment with algorithmic and digitally benchmarked academic evaluation environments.

Exposure to AI-assisted, algorithmic, and digitally benchmarked evaluation systems was assessed as a multiple-response variable. The full exposure-type distribution is reported in Appendix Table T6. Overall, the demographic distribution supports sample relevance because respondents had sufficient academic experience and institutional exposure to evaluate AI-assisted and digitally benchmarked academic evaluation systems.

### 4.1 Measurement model assessment

**Figure 2:**
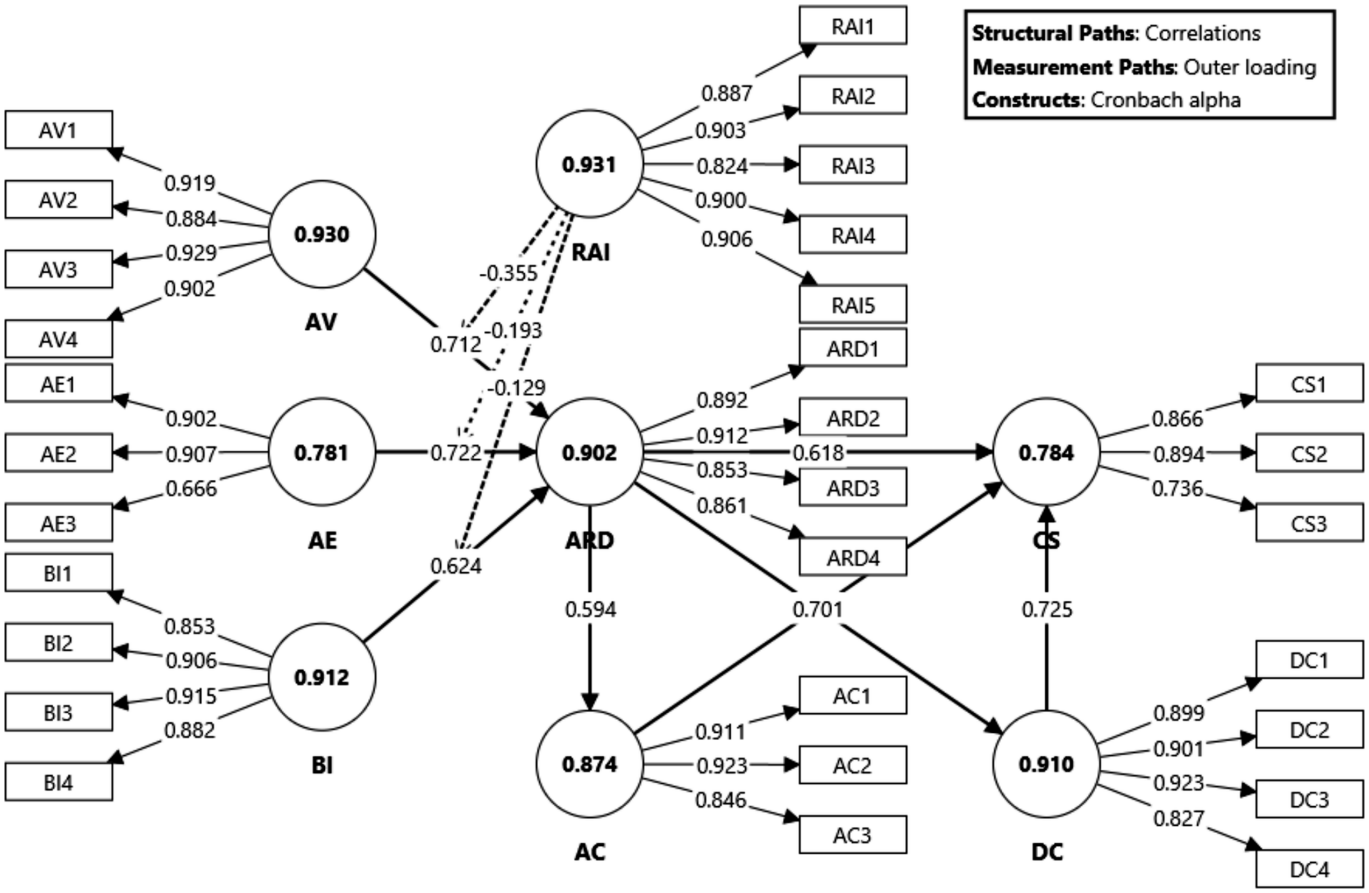
Measurement model.

The measurement model was evaluated using reflective PLS-SEM criteria, supplemented by exploratory factor evidence and CFA-style model comparisons to strengthen construct validation. This broader validation strategy was necessary because ARD is a focal construct and is conceptually close to related perceptions such as evaluation pressure, threat, identity disruption, and career insecurity. Following established measurement recommendations, the assessment considered indicator reliability, internal consistency reliability, convergent validity, multicollinearity, discriminant validity, and alternative measurement-model fit (Dijkstra and Henseler, 2015; Hair et al., 2022; Kline, 2016) (see Figure 2).

As reported in Table 4, PLS outer loadings were generally strong. Excluding AE3, the loadings ranged from 0.736 to 0.929. AE3 loaded at 0.666, which is below the conventional 0.70 guideline. However, loadings between 0.60 and 0.70 may be retained in adapted scales when construct-level reliability and AVE remain satisfactory and when the item contributes to content validity (Hair et al., 2022). AE3 was therefore retained because deleting it did not materially improve CR or AVE and because the item captures an important aspect of perceived explainability. EFA loadings also showed that items loaded primarily on their intended factors, with maximum cross-loadings below 0.30, suggesting no severe cross-loading problem and supporting the intended dimensional structure (Tabachnick and Fidell, 2019). CFA standardized loadings ranged from 0.651 to 0.918, providing additional item-level support for the measurement structure (Kline, 2016).

**Table 4:** Exploratory and confirmatory measurement validation.

Cons.	Item	EFA loading	Max cross-loading	PLS outer loading	VIF	CFA loading	*α*	ρA	CR	AVE
AC	AC1	0.884	0.221	0.911	2.675	0.895	0.874	0.886	0.923	0.799
	AC2	0.902	0.236	0.923	2.977	0.911				
	AC3	0.821	0.207	0.846	1.975	0.838				
AE	AE1	0.862	0.248	0.902	2.072	0.874	0.781	0.856	0.869	0.693
	AE2	0.876	0.263	0.907	2.165	0.889				
	AE3	0.637	0.291	0.666	1.331	0.651				
ARD	ARD1	0.863	0.244	0.892	3.104	0.879	0.902	0.903	0.932	0.774
	ARD2	0.884	0.251	0.912	3.536	0.898				
	ARD3	0.821	0.233	0.853	2.141	0.841				
	ARD4	0.837	0.239	0.861	2.364	0.852				
AV	AV1	0.892	0.266	0.919	3.579	0.905	0.930	0.932	0.950	0.826
	AV2	0.858	0.241	0.884	3.097	0.872				
	AV3	0.907	0.273	0.929	4.294	0.918				
	AV4	0.878	0.258	0.902	3.369	0.891				
BI	BI1	0.826	0.231	0.853	2.348	0.842	0.912	0.915	0.938	0.791
	BI2	0.879	0.247	0.906	3.345	0.895				
	BI3	0.891	0.256	0.915	3.557	0.904				
	BI4	0.852	0.238	0.882	2.671	0.868				
CS	CS1	0.824	0.213	0.866	1.805	0.842	0.784	0.820	0.873	0.697
	CS2	0.859	0.226	0.894	1.951	0.872				
	CS3	0.704	0.197	0.736	1.431	0.721				
DC	DC1	0.881	0.269	0.899	3.619	0.896	0.910	0.910	0.937	0.789
	DC2	0.874	0.258	0.901	3.201	0.889				
	DC3	0.903	0.281	0.923	4.156	0.916				
	DC4	0.781	0.229	0.827	1.905	0.798				
RAI	RAI1	0.846	0.251	0.887	3.454	0.866	0.931	0.943	0.947	0.782
	RAI2	0.872	0.267	0.903	3.922	0.889				
	RAI3	0.798	0.222	0.824	1.902	0.817				
	RAI4	0.864	0.271	0.900	4.212	0.884				
	RAI5	0.875	0.276	0.906	4.493	0.891				

Internal consistency was satisfactory across constructs. Cronbach’s alpha values ranged from 0.781 to 0.931, composite reliability rho_A values ranged from 0.820 to 0.943, and composite reliability rho_C values ranged from 0.869 to 0.950. These values exceed commonly recommended thresholds and indicate adequate reliability across constructs. Convergent validity was also supported, with AVE values ranging from 0.693 to 0.826, all above the 0.50 criterion, indicating that each construct explains more than half of the variance in its indicators (Fornell and Larcker, 1981). VIF values ranged from 1.331 to 4.493, below the conservative threshold of 5.0, suggesting no problematic indicator-level multicollinearity (Hair et al., 2022).

Discriminant validity was first assessed using the HTMT ratio, which is generally considered more sensitive than the Fornell–Larcker criterion for detecting lack of discriminant validity in variance-based SEM (Henseler et al., 2014). As shown in Table 5, all HTMT values were below the 0.90 threshold, and most were below 0.85. However, several values were relatively high, including BI–CS (0.826), DC–CS (0.841), AE–ARD (0.818), and AV–ARD (0.776). Therefore, discriminant validity was not inferred from threshold compliance alone. Instead, the HTMT evidence was interpreted together with the EFA pattern, CFA loadings, and alternative model comparisons, as recommended when constructs are theoretically adjacent and self-reported (Henseler et al., 2014; Voorhees et al., 2017).

**Table 5:** Discriminant validity (HTMT).

Construct	AC	AE	ARD	AV	BI	CS	DC	RAI
AC								
AE	0.210							
ARD	0.664	0.818						
AV	0.604	0.672	0.776					
BI	0.800	0.249	0.683	0.569				
CS	0.279	0.384	0.721	0.587	0.826			
DC	0.212	0.200	0.767	0.596	0.748	0.841		
RAI	0.210	0.546	0.521	0.666	0.555	0.565	0.547	

The supplementary CFA model comparisons reported in Appendix T2 provide additional evidence of construct distinctiveness. The proposed eight-factor model showed acceptable fit, *χ*^2^/df = 2.21, CFI = 0.956, TLI = 0.948, RMSEA = 0.052, and SRMR = 0.044. These values meet commonly accepted guidelines for acceptable-to-good model fit (Kline, 2016). The proposed model also fit better than all restrictive alternatives. The one-factor model showed poor fit, indicating that the data are not adequately represented by a single general factor. More importantly, models merging ARD with career sustainability, ARD with defensive reconfiguration, adaptive with defensive reconfiguration, and algorithmic visibility with explainability all produced weaker fit. These results support the empirical distinctiveness of the focal constructs, including ARD.

### 4.2 Predictive relevance assessment

Predictive relevance was assessed using the PLSpredict procedure and by comparing the PLS-SEM prediction errors with those of a linear regression benchmark model (LM), in line with recent PLS-SEM guidance (Alam et al., 2025a; Hair et al., 2022). As shown in Table 6, the Q^2^predict values for the career sustainability indicators were positive, indicating that the model has predictive relevance for these indicators. The Q^2^predict values ranged from 0.228 to 0.389, suggesting small-to-moderate predictive relevance for the career sustainability indicators.

**Table 6:** Predictive statistics.

MV	Q^2^predict	PLS-SEM_RMSE	LM_RMSE	IA_RMSE
CS1	0.389	0.818	0.717	1.046
CS2	0.380	0.870	0.704	1.104
CS3	0.228	0.939	0.878	1.069

However, the RMSE comparison shows that the LM benchmark produced lower prediction errors than the PLS-SEM model for all three career sustainability indicators. Therefore, although the positive Q^2^predict values indicate predictive relevance, the model does not demonstrate superior out-of-sample predictive performance relative to the simpler linear benchmark. These results should therefore be interpreted cautiously. The findings support the model’s explanatory relevance for theory testing, particularly the role of algorithmic relative devaluation and career reconfiguration mechanisms, but they do not provide strong evidence that the PLS-SEM specification offers incremental predictive advantage over a linear benchmark.

### 4.3 Hypothesis testing and discussion

The structural model explains substantial variance in algorithmic relative devaluation (ARD; *R*^2^ = 0.679), adaptive career reconfiguration (AC; *R*^2^ = 0.353), defensive career reconfiguration (DC; *R*^2^ = 0.491), and career sustainability (CS; *R*^2^ = 0.666). These values suggest that the model captures meaningful variation in how faculty interpret algorithmic and digitally benchmarked evaluation systems and how these perceptions relate to career responses. Given the self-reported, time-lagged design without autoregressive controls or experimental manipulation, the coefficients are interpreted as theoretically ordered associations rather than causal effects.

Algorithmic visibility is positively associated with ARD (*β* = 0.433, *p* < 0.001, *f*^2^ = 0.272), supporting H1a. This indicates that visible dashboards, rankings, and digital scores may act not only as evaluation tools but also as status-signaling mechanisms that define which forms of academic contribution are institutionally recognized. In a metric-intensive context such as Chinese higher education, visible indicators may sharpen comparative exposure while leaving less measurable contributions, such as mentoring, collegial service, and pedagogical judgment, comparatively under-recognized.

**Figure 3:**
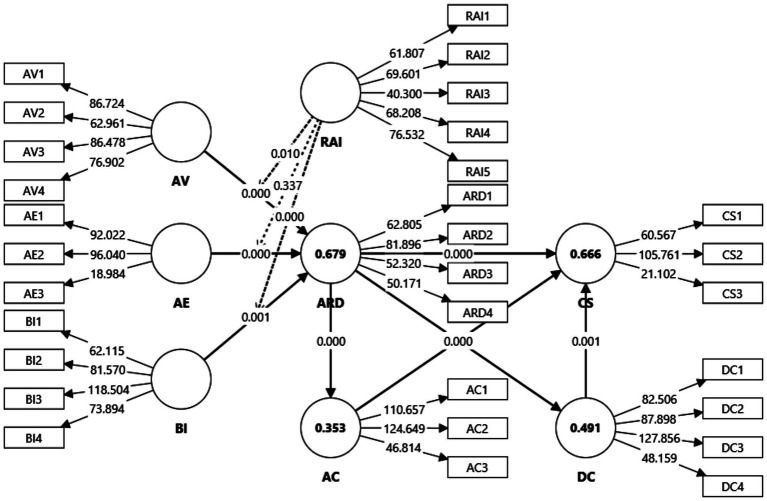
Structure model.

Benchmark intensity is also positively associated with ARD (*β* = 0.134, *p* = 0.001, *f*^2^ = 0.024), supporting H1b, although the effect is small. This suggests that continuous comparison against algorithmic or metricized standards contributes to perceived devaluation, but less strongly than the visibility of those standards. ARD therefore appears to reflect not only performance pressure, but also the perceived narrowing of professional recognition around visible and countable outputs (Figure 3).

Algorithmic explainability is negatively associated with ARD (*β* = −0.354, *p* < 0.001, *f*^2^ = 0.151), supporting H1c. Faculty who perceive algorithmic evaluation as more intelligible and reviewable are less likely to interpret it as devaluing their broader professional contribution. However, explainability should not be treated as a complete solution. It may reduce opacity and uncertainty, but it does not necessarily broaden what the evaluation system recognizes as valuable work.

The downstream paths show that ARD is positively associated with both adaptive career reconfiguration (*β* = 0.594, *p* < 0.001, *f*^2^ = 0.546) and defensive career reconfiguration (*β* = 0.701, *p* < 0.001, *f*^2^ = 0.965), supporting H2a and H2b. The large f^2^ values should be interpreted in light of the model structure: ARD is the sole direct substantive predictor of AC and DC in the main specification. Thus, these values reflect single-predictor equation effects rather than inflated incremental effects over multiple competing predictors. Substantively, the stronger ARD → DC path suggests that perceived devaluation may trigger protective distancing as well as proactive adaptation (Table 7).

**Table 7:** Structure model statistics.

Hypothesis path		*β*	*t*	*p*	95% CI	*f* ^2^	*R* ^2^
H1a	AV → ARD	0.433	7.779	<0.001	[0.346, 0.529]	0.272	0.679
H1b	BI → ARD	0.134	3.130	0.001	[0.066, 0.208]	0.024	—
H1c	AE → ARD	−0.354	6.543	<0.001	[−0.448, −0.269]	0.151	—
H2a	ARD → AC	0.594	17.675	<0.001	[0.535, 0.646]	0.546	0.353
H2b	ARD → DC	0.701	24.011	<0.001	[0.647, 0.744]	0.965	0.491
H2c	ARD → CS	−0.162	3.962	<0.001	[−0.230, −0.096]	0.039	0.666
H3a	AC → CS	0.546	11.284	<0.001	[0.464, 0.623]	0.350	—
H3b	DC → CS	−0.188	3.057	0.001	[−0.289, −0.087]	0.033	—
H4a	RAI × AV → ARD	−0.107	2.345	0.010	[−0.174, −0.024]	0.033	—
H4b	RAI × BI → ARD	0.145	4.690	<0.001	[0.098, 0.198]	0.045	—
H4c	RAI × AE → ARD	0.022	0.420	0.337	[−0.069, 0.105]	0.001	—
H5a	AV → ARD → AC	0.257	7.835	<0.001	[0.207, 0.315]	—	—
H5b	BI → ARD → AC	0.079	2.972	0.001	[0.039, 0.127]	—	—
H5c	AE → ARD → AC	−0.210	5.535	<0.001	[−0.279, −0.153]	—	—
H5d	AV → ARD → DC	0.303	8.214	<0.001	[0.245, 0.367]	—	—
H5e	BI → ARD → DC	0.094	3.065	0.001	[0.046, 0.147]	—	—
H5f	AE → ARD → DC	−0.248	5.861	<0.001	[−0.324, −0.184]	—	—
SI1	AV → ARD → AC → CS	0.140	6.158	<0.001	[0.107, 0.182]	—	—
SI2	BI → ARD → AC → CS	0.043	2.993	0.001	[0.022, 0.070]	—	—
SI3	AE → ARD → AC → CS	−0.115	5.346	<0.001	[−0.156, −0.084]	—	—
SI4	AV → ARD → DC → CS	−0.057	2.888	0.002	[−0.091, −0.027]	—	—
SI5	BI → ARD → DC → CS	−0.018	2.061	0.020	[−0.035, −0.007]	—	—
SI6	AE → ARD → DC → CS	0.047	2.753	0.003	[0.023, 0.080]	—	—
TE1	AV → CS	0.013	0.472	0.319	[−0.041, 0.068]	—	—
TE2	BI → CS	0.004	0.321	0.374	[−0.018, 0.026]	—	—
TE3	AE → CS	−0.011	0.401	0.344	[−0.064, 0.043]	—	—

*R*^2^ values are reported once for each endogenous construct. Confidence intervals are reported with signs aligned to the direction of the estimated coefficients. The relatively large *f*^2^ values for ARD → AC and ARD → DC reflect the single-predictor structure of the AC and DC equations and should be interpreted as single-predictor effect sizes rather than as incremental effects over multiple competing predictors.

ARD is negatively associated with career sustainability (*β* = −0.162, *p* < 0.001, *f*^2^ = 0.039), supporting H2c, although the effect is small. This indicates that perceived comparative downgrading may weaken confidence in long-term career continuity and viability. At the same time, the modest effect suggests that ARD does not automatically undermine career sustainability; its implications depend partly on how professionals respond.

Adaptive and defensive reconfiguration show opposite associations with career sustainability. Adaptive reconfiguration is positively related to CS (*β* = 0.546, *p* < 0.001, *f*^2^ = 0.350), supporting H3a, suggesting that skill development, digital tool integration, and expertise repositioning may help faculty remain viable in metricized evaluation systems. Defensive reconfiguration is negatively related to CS (*β* = −0.188, *p* = 0.001, *f*^2^ = 0.033), supporting H3b, indicating that distancing from metric-intensive or AI-exposed domains may protect professional identity in the short term but weaken perceived long-term career viability.

The moderation results indicate that responsible AI governance has an ambivalent role. Governance weakens the relationship between algorithmic visibility and ARD (*β* = −0.107, *p* = 0.010), supporting H4a. This suggests that visible metrics are less likely to be perceived as devaluing when embedded in procedures that allow transparency, fairness safeguards, participation, and contestability. However, governance strengthens the relationship between benchmark intensity and ARD (*β* = 0.145, *p* < 0.001), supporting H4b. This implies that in highly benchmarked regimes, formal governance may also legitimize intensive metric pressure when the underlying definition of valued contribution remains narrow. The interaction between governance and explainability is not significant (*β* = 0.022, *p* = 0.337), so H4c is not supported.

The mediation results support ARD as a proximal comparative-worth mechanism. Visibility and benchmark intensity have positive indirect effects on adaptive and defensive reconfiguration through ARD, while explainability has negative indirect effects through reduced ARD, supporting H5a–H5f. The serial indirect effects further show that ARD has ambivalent implications for career sustainability. Specifically, ARD may support career sustainability when it activates adaptive reconfiguration, but it may undermine career sustainability when it directly weakens perceived career viability or when it activates defensive reconfiguration.

The corrected total effects should be interpreted cautiously as model-implied aggregate associations. The aggregate associations of algorithmic visibility, benchmark intensity, and algorithmic explainability with career sustainability were very small and statistically non-significant under one-tailed tests: AV → CS (*β* = 0.013, *p* = 0.319), BI → CS (*β* = 0.004, *p* = 0.374), and AE → CS (*β* = −0.011, *p* = 0.344). These non-significant total effects do not contradict the significant indirect and serial indirect effects. Rather, they indicate that the model contains competing pathways with opposite signs. For visibility and benchmark intensity, the positive serial pathway through adaptive reconfiguration is largely offset by the negative direct pathway through ARD and the negative serial pathway through defensive reconfiguration. For explainability, the direction of the component pathways is reversed: explainability reduces ARD, which lowers adaptive reconfiguration but also reduces the direct and defensive devaluation pathways. In both cases, the opposing mechanisms nearly cancel one another at the aggregate level. Therefore, the findings do not support the interpretation that visibility or benchmark intensity has a meaningful positive total association with career sustainability, nor that explainability has a meaningful negative total association. Instead, the corrected total effects show that algorithmic evaluation features operate through significant but competing downstream pathways.

Overall, the findings support a restrained interpretation. Perceived algorithmic visibility and benchmark intensity are associated with higher ARD, while algorithmic explainability is associated with lower ARD. ARD is associated with both adaptive and defensive career reconfiguration, which have opposite implications for perceived career sustainability. Responsible AI governance weakens the devaluing association of visibility, strengthens the devaluing association of benchmark intensity, and does not significantly moderate the explainability–ARD relationship. The evidence does not show that AI-enabled evaluation objectively reorganizes professional value hierarchies; rather, it shows that perceived algorithmic evaluation features are linked to comparative devaluation appraisals and divergent career responses in a metric-intensive academic context.

## 5 Implications of this study

### 5.1 Theoretical implications

This study contributes to AI-work and career scholarship by showing that perceived algorithmic and digitally benchmarked evaluation features are associated with professionals’ perceptions of relative devaluation and career-related responses in a highly metricized academic context. Rather than claiming that AI-enabled evaluation objectively reorganizes professional hierarchies, the findings suggest a more bounded theoretical implication: when algorithmically visible standards become salient in evaluation routines, professionals may perceive that broader, less measurable forms of contribution receive weaker institutional recognition.

First, the study refines algorithmic management research by shifting attention from control and surveillance alone to perceived recognition within evaluation systems. Prior work has emphasized algorithmic monitoring, opacity, autonomy erosion, and worker control (Zhang et al., 2025). The present findings suggest that visibility may matter not only because it enables monitoring, but because it signals what the institution is prepared to notice and reward. The relatively stronger association between algorithmic visibility and ARD indicates that visible evaluation indicators may function as status-relevant signals, whereas benchmark intensity appears to exert a smaller direct effect. This distinction adds nuance to algorithmic management theory by separating evaluative pressure from the symbolic salience of visible metrics.

Second, the study positions ARD as a contextual refinement of existing constructs rather than as an entirely detached new concept. ARD overlaps with job insecurity, perceived unfairness, identity threat, and status threat, but its unique value lies in specifying a narrower appraisal: the perception that one’s broader contribution is comparatively downgraded when algorithmically visible standards become privileged evaluative referents. This helps explain why professionals may respond to AI-enabled evaluation even when immediate employment loss is not the primary concern. The contribution is therefore not that ARD replaces existing frameworks, but that it clarifies a comparative-worth mechanism that existing AI-threat and job-insecurity perspectives do not fully capture.

Third, the findings refine career sustainability theory by showing that perceived devaluation may be associated with divergent career responses. Adaptive reconfiguration was positively associated with career sustainability, whereas defensive reconfiguration was negatively associated with it. This distinction challenges the assumption that career reconfiguration is uniformly beneficial. In metricized academic settings, professionals may adapt by developing AI-related competencies and repositioning expertise, but they may also protect valued work by distancing themselves from metric-intensive domains. Sustainable careers in algorithmically mediated evaluation contexts may therefore depend not only on adaptability, but on whether adaptation preserves or narrows professional meaning.

Fourth, the governance findings complicate simple responsible-AI narratives. Responsible AI governance weakened the visibility–ARD relationship but strengthened the benchmark intensity–ARD relationship, while its interaction with explainability was not significant. This suggests that governance is not automatically protective. It may reduce devaluation when it makes visible metrics accountable and contestable, but it may also legitimize intensive benchmarking if it formalizes narrow indicators without broadening what counts as valuable contribution. The theoretical implication is that responsible AI governance should be understood not only as procedural control, but also as interpretive governance: it shapes how algorithmic standards are understood, justified, and institutionalized.

Overall, the study suggests that AI-enabled and digitally benchmarked evaluation may become career-relevant through perceived changes in recognition, not only through displacement anxiety, surveillance, or technostress. However, broader claims about AI restructuring professional value hierarchies should be treated as theoretical possibilities requiring further evidence, rather than conclusions fully established by the present data.

### 5.2 Practical implications

The findings have implications for universities and other metric-intensive professional organizations using AI-enabled or digitally benchmarked evaluation systems. The central managerial issue is not only whether algorithmic metrics are accurate or transparent, but whether they narrow the range of professional contribution that is recognized.

First, institutions should avoid allowing visible metrics to become the sole proxy for professional worth. Dashboards, rankings, citation indicators, teaching analytics, and grant metrics may improve administrative comparability, but they can also make less quantifiable contributions appear secondary. Evaluation systems should therefore combine quantitative indicators with qualitative review, peer judgment, mentoring contributions, service roles, pedagogical quality, and longer-term scholarly development.

Second, benchmarking intensity should be carefully governed. The findings suggest that continuous comparison may become devaluing when benchmarks are experienced as institutionally authoritative and difficult to contextualize. Institutions should clarify the scope, purpose, and limits of algorithmic or metricized benchmarks, and ensure that such indicators are not treated as exhaustive measures of academic value.

Third, explainability should be paired with meaningful contestability. Making algorithmic evaluation more understandable is useful, but transparency alone may not resolve devaluation if the underlying standards remain narrow. Faculty should have opportunities to question, contextualize, and supplement algorithmic outputs with evidence of contribution that is not easily captured by metrics.

Fourth, career support should distinguish adaptive from defensive reconfiguration. Institutions should not assume that all career adjustment reflects positive adaptation. Skill development, AI literacy, and digital tool integration may support sustainable careers, but withdrawal from metric-intensive domains may signal perceived misrecognition. Universities should therefore provide structured support for adaptive reconfiguration while also examining why some faculty feel compelled to protect their work from algorithmic evaluation.

Finally, responsible AI governance should preserve plural definitions of academic value. Governance should not merely make benchmarking more procedurally defensible; it should ensure that evaluation systems remain open to multiple forms of contribution. In this sense, responsible AI governance should address not only fairness and transparency, but also recognition.

### 5.3 Conclusion and future research

This study examined how perceived algorithmic and digitally benchmarked evaluation features are associated with algorithmic relative devaluation, career reconfiguration, and perceived career sustainability among faculty in Chinese higher education. The findings suggest that algorithmic visibility and benchmark intensity are positively associated with ARD, while algorithmic explainability is negatively associated with ARD. ARD, in turn, is associated with both adaptive and defensive career reconfiguration, which have opposite implications for perceived career sustainability.

These findings support a cautious conclusion: in highly metricized academic contexts, perceived algorithmic evaluation features may shape career-related perceptions through comparative devaluation appraisals. The evidence does not demonstrate that AI objectively redefines professional worth or restructures academic hierarchies. Rather, it shows that faculty perceptions of algorithmic evaluation are associated with perceived relative devaluation and differentiated career responses.

Future research should extend this work in several directions. First, longer longitudinal designs with baseline controls are needed to examine whether ARD changes over time and whether career reconfiguration produces durable sustainability outcomes. Second, experimental or quasi-experimental studies could isolate the effects of visibility, benchmark intensity, and explainability more precisely. Third, qualitative research could examine how professionals interpret algorithmic evaluation in everyday institutional practice, especially where quantitative indicators conflict with professional judgment. Fourth, cross-national and cross-sector studies could test whether ARD is specific to highly metricized academic systems or also appears in less metric-intensive professions. Finally, future research should unpack responsible AI governance into distinct components such as transparency, appealability, participation, distributive fairness, and recognition of non-metric contribution.

The broader theoretical possibility raised by this study is that AI-enabled evaluation may influence careers not only by changing tasks or skills, but by changing how professionals perceive the recognition of their contribution. Whether this becomes a wider restructuring of professional value hierarchies remains an important question for future research.

## Data Availability

The raw data supporting the conclusions of this article will be made available by the authors, without undue reservation.
